# Comparative assessment of the Willems dental age estimation methods: a Chinese population-based radiographic study

**DOI:** 10.1186/s12903-022-02418-5

**Published:** 2022-09-03

**Authors:** Jian Wang, Linfeng Fan, Shihui Shen, Meizhi Sui, Jiaxin Zhou, Xiaoyan Yuan, Yiwen Wu, Pingping Zhong, Fang Ji, Jiang Tao

**Affiliations:** 1 Department of General Dentistry, Shanghai Ninth People’s Hospital, Shanghai Jiao Tong University School of Medicine, College of Stomatology, Shanghai Jiao Tong University, Shanghai, China; 2 Department of Radiology, Shanghai Ninth People’s Hospital, Shanghai Jiao Tong University School of Medicine, College of Stomatology, Shanghai Jiao Tong University, Shanghai, China; 3 Department of Orthodontics, Shanghai Ninth People’s Hospital, Shanghai Jiao Tong University School of Medicine, College of Stomatology, Shanghai Jiao Tong University, Shanghai, China; 4 National Center for Stomatology, National Clinical Research Center for Oral Diseases, Shanghai Key Laboratory of Stomatology, Shanghai, China

**Keywords:** Dental age estimation, Willems method, Chinese population, Tooth development

## Abstract

**Background:**

The comparison of the two Willems dental age estimation methods (gender-specific (Willems I) and non-gender-specific (Willems II)) has not been fully investigated. Here we aimed to explore the applicability of the Willems dental age estimation in an Eastern Chinese population, which may cast light on the field of dental age estimation.

**Methods:**

A total of 1211 oral panoramic radiographs (582 boys and 629 girls) of the Chinese Han population aged 11–16 years old were collected. Dental ages (DAs) were calculated using the Willems method. Statistical significance was set at a p-value < 0.05. Age differences between chronological age (CA) and dental age were analyzed by paired t-tests and mean absolute error (MAE).

**Results:**

The differences between CA and DA determined by the Willems I method were + 0.44 and + 0.09 years for boys and girls, respectively. When using the Willems II method, these differences were + 0.57 and − 0.09. The MAEs of the Willems I method between DA and CA were 0.95 and 1.00 years in boys and girls, respectively. For Willems II, MAEs were 1.02 and 1.00 years in boys and girls.

**Conclusions:**

This study showed that the Willems I method was more accurate than the Willems II method in the boys’ group for predicting age from a whole scale. In comparison, Willems II is more competitive in the girls' group. Neither method may be satisfactory for 11-to-16-year-old teenagers in Eastern China.

## Introduction

The development characteristics of teeth have been applied in legal, forensic, and clinical fields as an effective weapon to decipher age information [1–3]. In the realm of legal, and forensic science, the decoded age information has cast light on many social affairs, such as welfare distribution, athlete selection, child enrollment, cadaver identification, and refugee checks [4, 5]. More importantly, age estimation helps juveniles delineate to a large extent for doubted age-related problems [6]. In the community of the clinic, applications have been extended to support routine therapeutic diagnosis and strategy decisions in orthodontics and pediatric dentistry [7].

To date, several radiographic dental age assessment methods for children have been proposed and validated, such as Demirjian’s [8], Nolla’s [9], Willems’s [10], Kvaal’s [11], Cameriere’s [12], Haavikko’s [13], and the London atlas [14], which adopt developing features of teeth reflected on radiographs. Taking Willems method for instance, it was first proposed by Willems et al. based on a Belgian-Caucasian population in 2001 [10]. The Willems method, in other words, a modified Demirjian dental age assessment method, simplified the former method by leaving out a step of data processing and testified its accuracy for dental age assessment in Europe. Inspired by Demirjian and his colleagues, the first Willems method remained a sex-specific trait, similar to the Demirjian method mentioned. Later, Willems et al. rethought the old method and constructed a reduced non-gender specific method (Willems II) to better resolve the problem of possible gender uncertainty under some scenarios [15]. Willems II method provides a common standard for both genders. It will display good performance in bioarchaeological studies under the circumstances that the sex of skeletal remains is unknown. Although studies on the Willems I method have been well documented on the planet, over- and underestimation of age have been announced in different parts of the world [1, 5, 9, 16–28]. To the best of our knowledge, the practical effect of the Willems II method has only been reported in Nemsi’s, Hedgel’s, and Urzi’s works and has not been fully investigated thus far [18, 29, 30]. With respect to Chinese populations, dental age estimations concerning the Willems I method have been reported over recent years [1, 4, 5, 19, 24, 31]. However, no Willems II method has been tested in China. Hence, the better choice of the two Willems methods applied in a population of eastern China is meaningful to explore. This work was designed to compare the applicability and accuracy of the two Willems dental age methods (Willems I vs Willems II) among children aged 11–16 years old in an eastern Chinese population.

## Methods and materials

### Methods

Retrospective cross-sectional research was conducted in a hospital in Shanghai, eastern China. The Independent Ethics Committee of the Shanghai Ninth People’s Hospital, affiliated with Shanghai Jiao Tong University School of Medicine (*2017–282-T212*) approved and authorized the project.

All the included orthopantomograms (OPGs) were randomly selected from the database of the hospital and followed by strict inclusion standards, which have been elucidated in previous work [7].

The patients underwent an OPG check before any oral treatments. All 1211 samples were from a Chinese Han population aged 11–16 years old.

### Data analysis

A total of 1211 OPGs were qualified for the current work. The information of each age group at an interval of one year is displayed in Table 1.

**Table 1:** Sample distribution by gender and age

Age group	Boys	Girls	Total
11	73	105	178
12	112	97	209
13	109	99	208
14	106	105	211
15	96	110	206
16	86	113	199
Total	582	629	1211

CA was calculated by the date of the OPG taken preoperatively minus the date of birth, which was expressed by two decimal points. All X-ray images were evaluated by the Willems method (Willems I and Willems II). To acquire the actual DA, we have to evaluate all the 7 left mandibular teeth (with third molars excluded)’s developmental stages (from A to H) judging from the status of the initial crown’s formation to the terminal apex’s closure. Then, we marked all the 7 teeth’s scores and summed them up to obtain the ideal DA value with the provided dataset tables.

Cohen’s kappa tests were employed to make the inner- and inter-agreements’ tests [32]. Intra- and inter-agreements were calculated to give better quantitative values of agreements during the studies with the repeated data. The analysis included descriptive and inferential statistics. Descriptive statistics such as the mean differences and standard deviation (SD) were calculated. Differences between DA and CA were generated by subtracting DA from CA, i.e., (DA-CA). Then, differences were stratified based on age and sex and analyzed using the paired *t-test*. The mean absolute error (MAE) was used to assess the precision of the two Willems methods. All analyses were conducted in SPSS 17.0 for Windows (SPSS, Inc., Chicago, IL). A p-value less than 0.05 was considered to be statistically significant.

## Results

A total of 1211 OPTs of sub-adults aged from 11 to 16 years were eligible for the present study, including 582 boys and 629 girls. The samples were classified into 6 subgroups at an interval of one year. Details concerning samples were listed in Table 1.

### Inter and intra-agreements

The Cohen’s kappa values reached 0.78 and 0.79 for boys and girls, respectively, which displayed good inter-and intra-agreement in the experiments.

### Willems I method

**Fig. 1:**
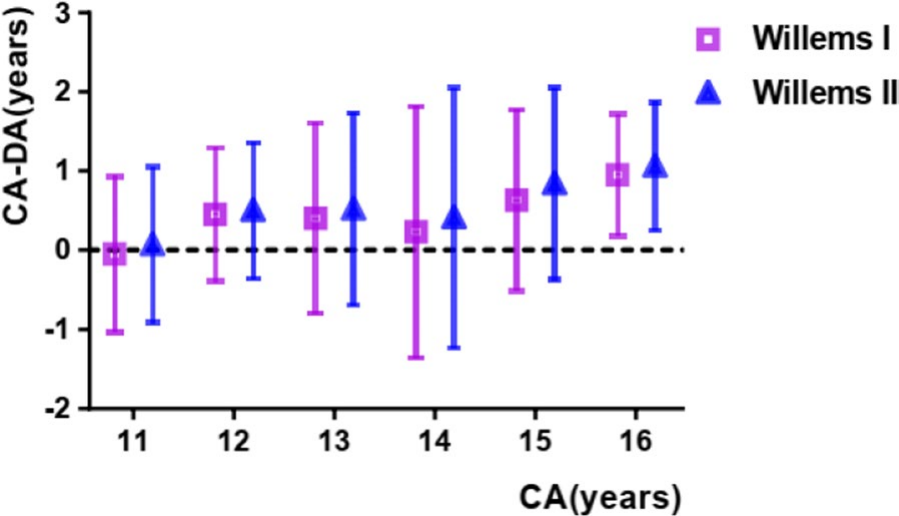
Willems I versus Willems II in boys group

**Fig. 2:**
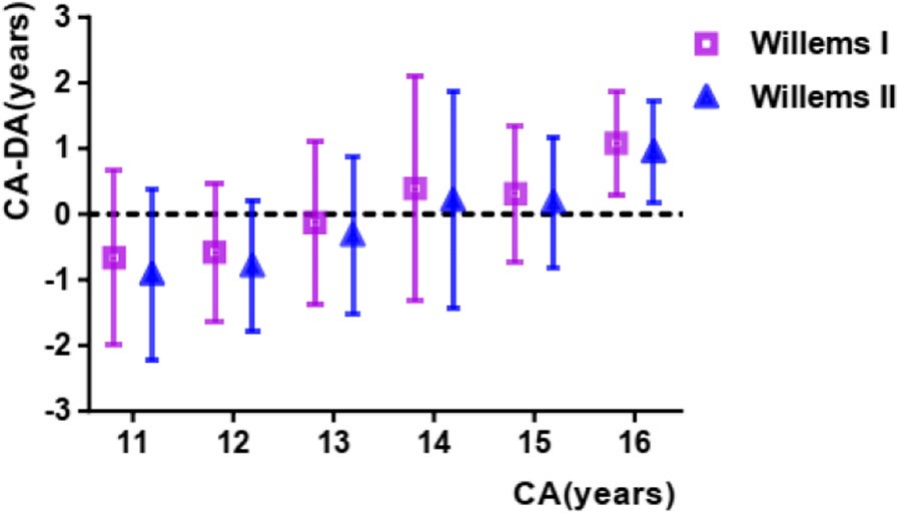
Willems I versus Willems II in girls group

Using the Willems I dataset, the mean dental age for boys and girls was 13.54 and 13.93 years, respectively (Tables 2 and 4). The general mean difference (CA-DA) for boys and girls was 0.44 ± 1.17 (p < 0.05) and 0.09 ± 1.37 (p > 0.05), respectively. An underestimation (CA-DA) of age was observed in all the subgroups except for boys in the 11–12-year group and girls in the 11–12, 12–13, and 13–14-year groups. The mean absolute error was 0.95 and 1.00 years for boys and girls, respectively (seen in Figs. 1 and 2).

**Table 2:** Accuracy comparison of Willems I method of dental age estimation

Gender	Age group	CA (years) (mean ± SD)	DA (years) (mean ± SD)	CA–DA (years) (mean ± SD)	p	MAE
Boys	11	11.47 ± 0.28	11.52 ± 0.91	(−)0.05 ± 0.98	0.665	0.78
	12	12.50 ± 0.28	12.05 ± 0.83	0.45 ± 0.84	0	0.75
	13	13.46 ± 0.28	13.05 ± 1.18	0.40 ± 1.20	0.001	1.03
	14	14.42 ± 0.31	14.19 ± 1.60	0.23 ± 1.59	0.147	1.12
	15	15.48 ± 0.28	14.85 ± 1.15	0.63 ± 1.14	0	1.05
	16	16.48 ± 0.24	15.53 ± 0.73	0.95 ± 0.77	0	0.95
	Total	13.98 ± 1.63	13.54 ± 1.79	0.44 ± 1.17	0	0.95
Girls	11	11.45 ± 1.16	12.11 ± 1.64	(−)0.66 ± 1.34	0	1.03
	12	12.46 ± 0.29	13.03 ± 1.04	(−)0.58 ± 1.05	0	0.86
	13	13.49 ± 0.26	13.62 ± 1.26	(−)0.13 ± 1.24	0.289	1.02
	14	14.47 ± 0.32	14.09 ± 1.75	0.39 ± 1.72	0.025	1.25
	15	15.43 ± 0.27	15.12 ± 1.04	0.31 ± 1.04	0	0.76
	16	16.46 ± 0.29	15.38 ± 0.82	1.08 ± 0.80	0	1.08
	Total	14.03 ± 1.81	13.93 ± 1.73	0.09 ± 1.37	0..091	1

### Willems II method

Adopting the Willems II method, the mean dental age for boys and girls was 13.41 and 14.11 years, respectively (Tables 3 and 4). The overall mean difference (CA-DA) for boys and girls was 0.57 ± 1.20 (p < 0.05) and (-)0.09 + 1.34 (p > 0.05), respectively. As can be reflected in Figs. 1 and 2, an underestimation (CA-DA) of age was observed in all the subgroups except for the girls in the 11–12, 12–13, and 13–14 years groups. The mean absolute error was 1.02 and 1.00 years for boys and girls, respectively (seen in Figs. 1 and 2).

**Table 3:** Accuracy comparison of Willems II method of dental age estimation

Gender	Age group	CA (years) (mean ± SD)	DA (years) (mean ± SD)	CA–DA (years) (mean ± SD)	p	MAE
Boys	11	11.47 ± 0.28	11.4 ± 0.91	0.07 ± 0.98	0.556	0.8
	12	12.50 ± 0.28	11.99 ± 0.84	0.5 ± 0.86	0	0.78
	13	13.46 ± 0.28	12.94 ± 1.20	0.52 ± 1.21	0	1.07
	14	14.42 ± 0.31	14.01 ± 1.66	0.41 ± 1.64	0.012	1.24
	15	15.48 ± 0.28	14.64 ± 1.24	0.84 ± 1.21	0	1.14
	16	16.48 ± 0.24	15.41 ± 0.77	1.06 ± 0.81	0	1.06
	Total	13.98 ± 1.63	13.41 ± 1.78	0.57 ± 1.20	0	1.02
Girls	11	11.45 ± 1.16	12.35 ± 1.58	(−)0.91 ± 1.30	0	1.01
	12	12.46 ± 0.29	13.25 ± 0.99	(−)0.79 ± 1.00	0	0.94
	13	13.49 ± 0.26	13.81 ± 1.21	(−)0.32 ± 1.19	0.008	0.75
	14	14.47 ± 0.32	14.26 ± 1.68	0.21 ± 1.65	0.205	1.17
	15	15.43 ± 0.27	15.25 ± 0.99	0.18 ± 1.00	0.061	0.75
	16	16.46 ± 0.29	15.50 ± 0.79	0.95 ± 0.77	0	0.95
	Total	14.03 ± 1.81	14.11 ± 1.66	(−)0.09 + 1.34	0.106	1

**Table 4:** Comparison of accuracy of Willems I and II methods

Gender	N	Willems I		Willems II		Willems I vs II	
		CA-DA (mean ± sd)	MAE	CA-DA (mean ± sd)	MAE	I-II (mean ± sd)	p-value
Boys	582	0.44 ± 1.17	0.95	0.57 ± 1.20	1.02	(−)0.13 ± 0.23	0
Girls	629	0.09 ± 1.37	1.00	(−)0.09 ± 1.34	1	0.18 ± 0.08	0
Total	1211	0.26 ± 1.28	0.98	0.23 ± 1.32	1.01	0.03 ± 0.23	0

## Discussion

The Willems method has gained popularity to a great extent as an easy-to-check approach for estimating the actual age. The Willems I method’s over/underestimation of age has been well reported worldwide. overestimation of chronological age was validated in a variety of counties or regions such as India [33],Turkey [34], Thailand [35], Spain [36],Kenya [37] and Poland [38]. Elsewhere, there were also reports of underestimation in north China [24], Tunisia [30], and Sri Lanka [39]. In allusion to the differences of the Willems I method among several regions, a systemic review, and meta-analysis we conducted previously concerning the Willems I method revealed the Willems method overestimated CA by 0.18 years and 0.06 years for boys and girls, respectively [4]. It also revealed that ethnicity specificity was necessary when adopting the Willems I method. This conclusion was consistent with other similar meta-analyses [25, 26, 40]. In the current work, the underestimation of age was + 0.44 ± 1.17 (p < 0.05) and + 0.09 ± 1.37 (p = 0.091) for the boy and girl groups, respectively. From the whole perspective, the girls’ group showed a more accurate effect of age assessment when compared with the boys’ group. This phenomenon was also viewed in our previous works and other printed studies [5, 41]. In subgroups’ accuracy analysis, the Willems I method was acceptable in 11–12, 12–13,13–14, and 14–15 years group of boys, while in 13–14, 14–15 and 15–16 years groups of girls, relatively compatible intervals were observed for age assessment.

In the journey of investigating the Willems II method’s applicability, few works have been reported over the last decades (Table 4). Urzel et al. observed a mean difference between CA and DA of 0.03 and 0.00 years for both genders with the Willems I and II method, respectively [15]. They concluded that both methods were appropriate when applied to a French population, although the Willems I method was more accurate according to their data. Another similar study from an Indian population with the Willems II method revealed that the mean values were 0.06 ± 0.80, − 0.11 ± 0.79, and − 0.01 ± 0.80 years for boys, girls, and the total sample, respectively [33]. Among Tunisian sub-adults [34], the Willems II method tended to underestimate age by 0.91, and 0.64 years for boys and girls, respectively. Whereas the Willems I method indicated an underestimation of chronological age by 0.40 years for boys, and by 0.69 years for girls. In their investigation, Willems I was more accurate when compared with Willems II in the Tunisian population. In the current study, the mean values of CA-DA were 0.57 ± 1.20 (p < 0.05) and − 0.09 + 1.34 (p = 0.106) years for boys and girls, respectively. A more accurate estimation was observed in the girls’ group in both methods. These results revealed that Willems I was more accurate and reliable for boys, while Willems II was a better option for girls. Our results are in accordance with the aforementioned studies. To be more specific, Willems I or II was an optional choice for ascertaining age despite Willems I having a slightly higher accuracy for 11–12, 12–13, 1, 3-, 14 and 14–15 years groups among boys according to our data. In girls’ subgroups, the data appeared to confirm that Willems II lost its advantages in younger age groups of 11–12 and 12–13 years, and regained its leading positions in 13–14 and 14–15 years, groups. The results showed that neither method was a reliable choice for evaluating 16–17 years old for both genders. From our’s perspective, when an individual grows 16 years or older, the apex’s closure has been completed in most cases, we could not tell the more differences from the radiograph via Willems method. The inner drawbacks posed restrictions on individuals of age large than 16 years old for assessing dental age via the Willems method.

Despite the accuracy assessment of Willems I and Willems II, the study still has limitations that must be elucidated. The samples we collected ranged only from 11 to 16 years old, we did not retrieve lower age groups younger than 11 years old. In our initial design of the project, we intended to take a clear and accurate evaluation of some important age thresholds like 14 and 16 years old. Because these thresholds provide key information to verdict a teenager’s delinquency in China. We have to admit that if we could have recruited individuals less than 11 years old, our research would have a deep and comprehensive understanding of the two Willems methods. Despite we have performed the dental age evaluation among 11–16-year-old sub-adults, more age subgroups will be recruited and show up in our subsequent works.

## Conclusions

In conclusion, the present study investigated the applicability of the Willems I and Willems II methods with a sample of Eastern Chinese teenagers aged 11–16 years old. These outcomes suggest that Willems I is more accurate for boys, while the Willems II method prefers girls; Neither method may be a perfect match for the Eastern Chinese Han population despite some subgroups having clear and accurate age assessment. An ethnicity-specific model based on the Willems method or further modifications is encouraged to prosper the science of dental age estimation.

## Data Availability

The datasets used for the present study are available from the corresponding author on reasonable request.

## Abbreviations

DA: Dental age
CA: Chronological age
MAE: Mean absolute error
SD: Standard deviation
N: Number
OPG: Orthopantomogram
